# The Philosophy of Mastication

**Published:** 1913-04-15

**Authors:** George M. Niles

**Affiliations:** Atlanta, Ga.


### THE PHILOSOPHY OF MASTICATION.

GEORGE M. NILES, M.D., ATLANTA, GA.

The chewing of our food is a subject of more or less interest to all of us. Beginning with the precept inculcated in every nursery, we are constantly admonished throughout life that thorough mastication is a pre-requisite to health; while a rather recent school of thought contends that the whole process of bodily nutrition is markedly affected by the preliminary treatment of food in the mouth.

Mastication is an entirely voluntary act, while the performance of swallowing is a complicated reflex movement, which may be initiated voluntarily, but is, for the most part, completed independently of the will. Under normal conditions the presence of moist food on the tongue seems essential to the completion of this act, and I might add that a pleasant taste, coupled with a favorable mental attitude, still further facilitates the passage of food down the esophagus.

Too rapid eating, or tachyphagia, is a frequent fault, and has no doubt caused many digestive qualms, besides being the starting-point of many chronic disorders of the alimentary tract. This I admit. What I do *not* admit, however, is the necessity for slow, deliberate and systematic masticatication as a *sine qua non* for health in every individual, irrespective of temperament or station in life; nor do I believe it conducive to the best work of the digestive organs that a hard-and-fast rule be enjoined, whereby a certain stated period must be devoted to the mastication of a meal, regardless of the pleasure of the masticator.

From time to time apostles of deliberate mastication, or bradyphagia, have appeared on the horizon, the most prominent of these being Mr. Horace Fletcher, whose work, "The A. B. Z. of Our Nutrition," is so largely devoted to this topic, and who so well pleads its cause, that slow eating has come to be called "Fletcherism"; and to chew and insalivate food until it liquifies in the mouth is to "Fletcherize" it, according to the nomenclature.

Mr. Fletcher is an American, who, when middle-aged, obese, dyspeptic and discouraged, discovered that by slow and deliberate eating his health improved, and began to elaborate this supposedly new principle. Asserting that he literally chewed himself back into health, he also argued that by the extreme mastication and insalivation of food, appetite is satisfied with a much smaller amount than is ordinarily craved, while at the same time bodily and mental well-being is greatly enhanced.

Mr. Fletcher was really anticipated by Mr. Gladstone, who, as the first advocate of this fad in England, attributed much of his success in public life to the fact that he had always made it a rule to give every tooth a chance, counting thirty-two bites for each morsel.

Under Mr. Fletcher's skilled exploitation, mastication as an art has grown and budded and blossomed until it has become a new theory, so popular that any one seeking to impede its continued fruition is considered by many either an iconoclast or an ass.

As regards the protein constituents of food, insalivation exerts but little effect. We well know that either the pepsin and hydrochloric acid in the stomach or the trypsin beyond will attend to them, if they are decently comminuted, and their stay in the mouth need be only long enough to originate those psychic impulses which Pawlow has shown us regulate the subsequent flow of digestive juices. Carnivorous animals and reptiles habitually bolt their food, and zoologic history furnishes no record of any psychic forms of dyspepsia in these creatures. The essence of salivary digestion is the transformation of starch into sugar by the action of the ptyalin, and that process, though inauguarted in the mouth, continues until the whole of the stomach contents has become acid. The time of salivary digestion is brief, and to be effectual should be energetic. No more should be expected of it than a preliminary act. The pancreatic and other juices beyond the stomach will care for the starches, if only the psychic centers forward the tidings as received by the gustatory senses.

As to the method or comparative rapidity of chewing, I might say, and say correctly, it is to a great extent temperamental. As some people can perform a stated task, and perform it well in half the time required by slow-moving individuals; and as some people move quickly, speak quickly, and think quickly, so they also chew quickly, but well. By those ardent and strenuous spirits who are happiest when in the busy turmoil of competitive struggle, the act of mastication is naturally performed briskly, but none the less adequately. To that other class, who desire to meander through life in a leisurely way, "far from the madding crowd's ignoble strife," to those semivaletudinarians whose gastronomic powers are under constant mental scrutiny, fletcherism promises the fountain of youth.

Another objection to interminable mastication is the brevity of life. In one place Mr. Fletcher relates that one-fifth of an ounce of a young onion required 722 chews before it disappeared through involuntary swallowing, and Dr. Kellogg mentions a patient who cheerfully spent never less than an hour and a half in masticating his one small daily meal. To insist that busy men, those whose shoulders and minds bear the burdens and cares of government, commerce or literature; whose eager intellects are conquering the earth, the sea and the air—to insist that these should be subjected to a wearisome mastication of inanimate food, is a delusion and a snare, an anachronism in our twentieth-century civilization, and a frittering away of priceless time.

A short while ago there consulted me a cadaverous-looking dyspeptic, who informed me that, up to his retirement from active business five years ago, he had never experienced a digestive discomfort. During his laborious years he ate his breakfast hurriedly, so as to get to the store betimes. His lunch was snatched at a nearby restaurant while his evening meal was frequently rushed by some important engagement. The finer details of mastication never entered his head, nor did he realize that he was "digging his grave with his teeth," until so informed by an over-zealous friend acquired in his new life of leisure. The small seed, once sown, took root in his idle mind, and, with little else to do, he devoted himself assiduously to Fletchrizing his food and safeguarding his health. The result of this was a morbid introspection, which transformed a robust alert business man into a puny, whining invalid, full of pains and obsessions, and with every waking thought short-circuited on his stomach. That is not an uncommon case any observant physician will testify.

The students of digestion and nutrition, guided by the beacon lights furnished by Pawlow and Cannon, are each year according more and more potency to the psychic factors involved in every function of those organs, and benighted indeed is the intellect that can comprehend only the mechanic and chemic aspects of these all-important questions. When an appetizing meal is placed attractively before a hungry diner, both the eyes and the olfactories set in motion the preliminaries to the digestion of that meal. As each mouthful is received by the waiting gustatory tract, it is chewed and insalivated just enough to promote easy deglutition, and to allow its savory taste and aroma to be thoroughly appreciated. In the meanwhile numerous messages are being sent to the stomach, pancreas and other organs concerned, informing them in language perfectly intelligible how much and what kind of aliment is being sent them to digest. Should the messages be of cheerful import, these organs set to their task with alacrity, performing them with such ease and dispatch, that the fortunate owner need not know that he even owns a stomach.

On the other hand, should the same meal be taken with severe gastronomic contemplation, and with arduous and lengthy mastication, the message may be "flashed" to the same waiting organs: "Your master has lost confidence in you, the mouth and teeth will take over the most of your former duties, and hereafter you will be under constant mental surveillance." It is easily understood that under such conditions digestion is performed in a halting, complaining and unsatisfactory manner, and well may we say that "the last state of that man is worse that the first."

A certain amount of necessary chewing is advisable, as evidenced by the confirmation of the human teeth. If the chemical functions of the stomach are deficient, or if the outlet is greatly constricted, fine mastication and thorough insalivation are highly beneficial. Fletcherism is also allowable in those lackadaisical creatures whose tastes are gently epicurean, and who possess the desires of a Lucullus, minus the initiative. Let these harmless souls chew to their heart's content, for they need some fad, and this one can harm no one unless it be themselves.

To the great majority of every-day Americans, a simple injunction as to good teeth and ample mastication is entirely sufficient. But to those ardent beings who move impetuously through the drama of life, whose every act is intense and every movement like a flash—to these fletcherism is a thorn in the flesh, causing them to worry and chafe like a spirited horse held back by a cruel bit.

Each division of the alimentary tract has its rightful duties in the life-scheme of nutrition, and the magnification of any one is liable to prove an opening wedge for various digestive ills, that, like jealousy, furnish the food they feed and grow on.

As we cannot by thought add one cubit to our stature, so let me close with the paraphrase, "No man can by taking thought add energy to his stomach."—*Journal A. M. A.*, March 29, 1913.

